# Sustained Release of Melatonin from GelMA Liposomes Reduced Osteoblast Apoptosis and Improved Implant Osseointegration in Osteoporosis

**DOI:** 10.1155/2020/6797154

**Published:** 2020-05-28

**Authors:** Long Xiao, Jiayi Lin, Ruoyu Chen, Yu Huang, Yu Liu, Jiaxiang Bai, Gaoran Ge, Xiaopeng Shi, Yong Chen, Jiandong Shi, Lu Aiqing, Huilin Yang, Dechun Geng, Zhirong Wang

**Affiliations:** ^1^Department of Orthopedics, Zhangjiagang TCM Hospital Affiliated to Nanjing University of Chinese Medicine, Zhangjiagang 215600, China; ^2^Department of Orthopaedics, The First Affiliated Hospital of Soochow University, Suzhou 215006, China; ^3^Department of Gynecology, The First People's Hospital of Zhangjiagang, Soochow University, Zhangjiagang 215600, China; ^4^Department of Gynecology, The First Affiliated Hospital of Soochow University, Suzhou 215006, China

## Abstract

A reduction in bone mass around an implant is the main cause of implant loosening, especially in postmenopausal osteoporosis patients. In osteoporosis, excessive oxidative stress, resulting in osteoblast apoptosis, largely contributes to abnormal bone remodeling. Melatonin (MT) synthesized by the pineal gland promotes osteoblast differentiation and bone formation and has been effectively used to combat oxidative stress. Therefore, we hypothesized that MT attenuates osteoblast apoptosis induced by oxidative stress, promotes osteogenesis in osteoporosis, and improves bone mass around prostheses. Moreover, considering the distribution and metabolism of MT, its systemic administration would require a large amount of MT, increasing the probability of drug side effects, so the local administration of MT is more effective than its systemic administration. In this study, we constructed a composite adhesive hydrogel system (GelMA-DOPA@MT) to bring about sustained MT release in a local area. Additionally, MT-reduced apoptosis caused by hydrogen peroxide- (H_2_O_2_-) induced oxidative stress and restored the osteogenic potential of MC3T3-E1 cells. Furthermore, apoptosis in osteoblasts around the implant was significantly attenuated, and increased bone mass around the implant was observed in ovariectomized (OVX) rats treated with this composite system. In conclusion, our results show that GelMA-DOPA@MT can inhibit osteoblast apoptosis caused by oxidative stress, thereby promoting osteogenesis and improving bone quality around a prosthesis. Therefore, this system of local, sustained MT release is a suitable candidate to address implant loosening in patients with osteoporosis.

## 1. Introduction

The population in today's society is aging. Among health issues brought about by aging, osteoporosis has become one of the most serious issues and attracted the attention of orthopedist [1–3]. Osteoporosis is a metabolic disease characterized by bone mass loss and bone microstructure destruction, leading to fragility of the bone and increased bone fracture risk [4, 5]. Osteoporosis caused by aging brings about a chain reaction of effects; after implantation, internal fixation screws and pedicle screws exhibit implantation loosening to different extents, which leads to implantation failure [6–10]. Therefore, improving the stability of internal fixation under the pathological conditions of osteoporosis has become the focus and challenge of orthopedic clinical research.

At present, the use of screws with an enlarged diameter, traditional antiosteoporosis drugs, and bone cement injection are the main treatment methods in clinic [11–13]. Among these methods, systemic antiosteoporosis drugs, such as bisphosphonates, denosumab, raloxifene, and teriparatide, are used to strengthen the strength of osteoporotic bone internal fixation [14, 15]. As shown through clinical observation, this method can increase bone density in the whole body, but because long-term drug treatment is required and the drug needs to reach the site of action through systemic circulation, the local osteoporotic state of the implant is not significantly improved [16, 17]. In addition, by increasing the diameter and length of internal fixation screws, internal fixation in the osteoporotic vertebral body can be strengthened, increasing its efficacy [18, 19]. In severe osteoporosis patients, pedicle burst fracture occurs easily when the diameter of the screw exceeds 70% of the crosssectional area of the pedicle, so this method cannot meet the clinical needs of these patients.

Melatonin (MT) is an important steroid hormone secreted by the pineal gland with extensive clinical applications [20]. Specifically, MT is currently widely utilized to regulate various functions, such as the biological rhythm and immune system, and exerts antiaging, antioxidation, and antitumor effects [21–24]. Increasing evidence indicates that the production of oxidants and the cellular response to oxidative stress are intricately connected to the fate of implanted biomaterials. It has been demonstrated that osteoporosis-mediated accumulation of reactive oxygen species (ROS) may deleteriously affect the bone regeneration, leading to compromised implant osteointegration [25]. Moreover, recent studies on the influence of MT on hard tissues, such as bone and teeth, have attracted much attention [26, 27]. According to these studies, MT plays an important role in regulating bone formation and bone growth, shows potential in promoting bone differentiation, and is also able to reduce apoptosis caused by oxidative stress, which exerts antiosteoporosis effects [26, 28–30]. Moreover, considering the distribution and metabolism of MT, its systemic administration requires a large amount of the drug, increasing the probability of drug side effects, so the local administration of MT is more effective than its systemic administration. In addition, there have been few in vivo experiments on the use of MT locally at implants. Thus, for MT to exert antiosteoporosis effects at local implantation areas, it must be continuously and steadily released over the long term and in situ.

**Scheme 1:**
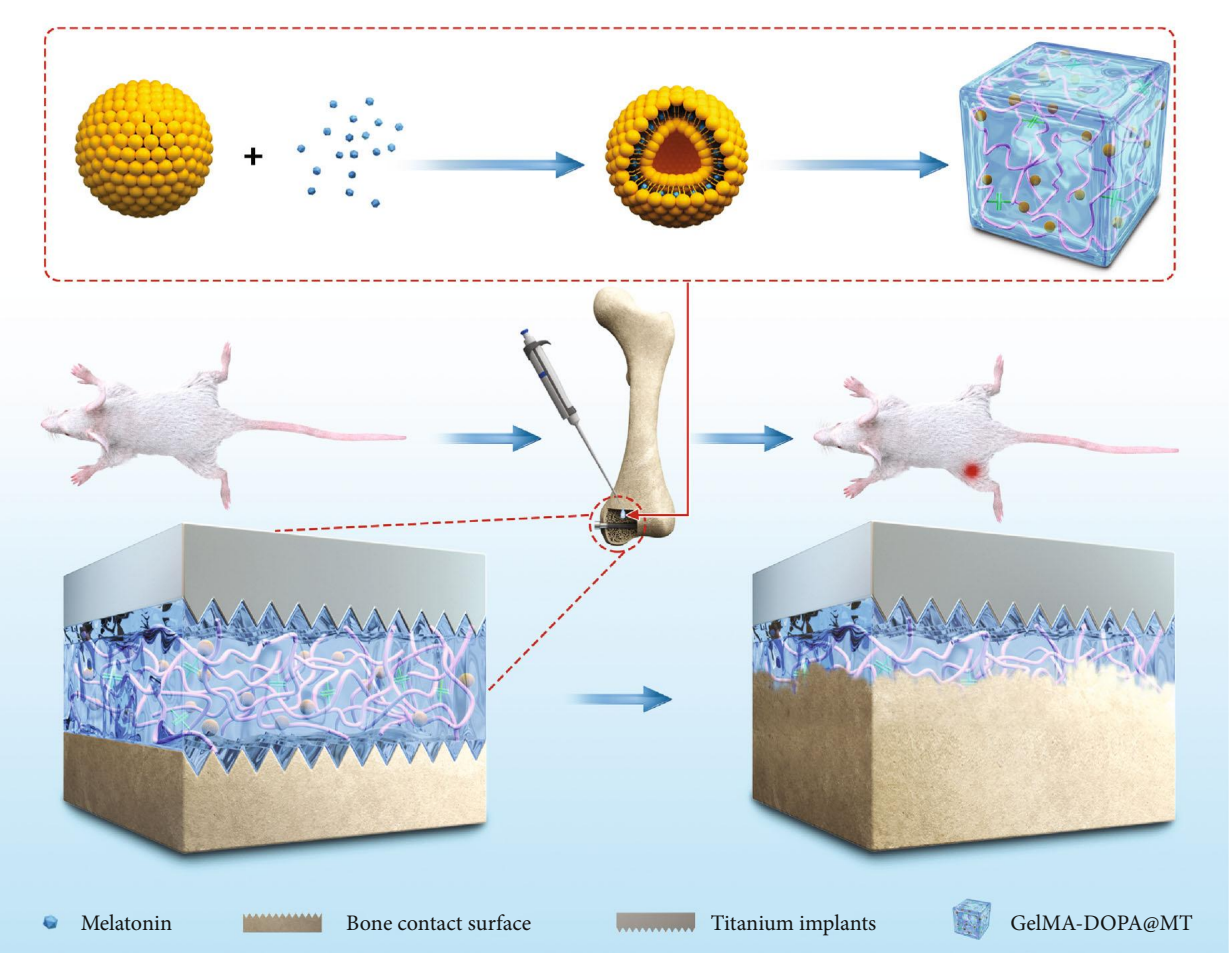
Schematic diagram: the mechanism by which GelMA-DOPA@MT promotes bone regeneration. The continuous melatonin release system is injected around the site where Ti implants are inserted into the bone. This system gradually degrades and releases melatonin, promoting the formation of the new bone around Ti implants.

Owing to its excellent adhesive ability and biocompatibility, gelatin methacryloyl-dopamine (GelMA-DOPA) has been widely used in bone tissue engineering [31–33]. Liposomes, which are well-known for their excellent biocompatibility and satisfactory ability to control drug release, have been widely utilized in various types of drug delivery [34–36]. In this study, we combined MT with GelMA-DOPA, which has potential adhesive functions on wet surfaces, to fabricate a composite implantation system to induce implant osseointegration in an osteoporotic state by reducing osteoblast apoptosis (Scheme 1).

## 2. Materials and Methods

### 2.1. Fabrication and Characterization of GelMA-DOPA

#### 2.1.1. Fabrication of GelMA-COOH

GelMA was fabricated as previously reported [37]. Briefly, 4 g of GelMA was dissolved in 80 mL of PBS in a flask by stirring (200 rpm) at 50°C. Then, 2 mL of triethylamine and 2 g of succinic anhydride in 40 mL of DMSO were added to the GelMA mixture. The resulting mixture was stirred overnight at 50°C under the protection of Ar, diluted in 200 mL of PBS, and neutralized with 0.1 M HCl. The product was poured into a dialysis bag (MW = 3500) and dialyzed for approximately one week. The resulting liquid was frozen at -80°C overnight and then lyophilized to obtain GelMA-COOH.

#### 2.1.2. Fabrication of GelMA-DOPA

Catechol motifs are key factors for the successful construction of adhesive materials. To conjugate catechol motifs to GelMA [38], 1 g of GelMA-COOH was dissolved in 10 mL of MES buffer (50 mM, pH 5), and the resulting solution was ultrasonicated for 30 min to remove any air bubbles. Then, 0.2 g of EDC, 0.3 g of NHS, and 0.2 g of dopamine hydrochloride were added to the solution. This whole process was carried out under the protection of Ar, and the mixture was stirred overnight at 25°C. Then, the solution was dialyzed against 0.01 M HCl in deionized water utilizing a dialysis bag (MW = 3500) for 4 days, followed by neutralization with 0.01 M NaOH. The resulting liquid was frozen at -80°C overnight and then lyophilized to obtain GelMA-DOPA.

### 2.2. Preparation and Characterization of Hydrogel

#### 2.2.1. Preparation of Hydrogel

GelMA, GelMA-COOH, and GelMA-DOPA were dissolved in PBS at concentrations of 20% (*w*/*v*), and 2-hydroxy-4-(2-hydroxyethoxy)-2-methylpropiophenone was added to the solution at 1% (*w*/*v*) as a photoinitiator [39]. In addition, GelMA-DOPA was dissolved in PBS at different concentrations ranging from 5% to 20%, and 1% photoinitiator was added to the liquid. The abovementioned precrosslinking solutions were poured separately into a custom-made round mold with a diameter of 15 mm and a depth of 5 mm and then photocrosslinked by ultraviolet (UV) light at 6.9 mW/cm^2^ d (360-480 nm) for 10 sec. Composite hydrogels with different concentrations (5-20%) of GelMA-DOPA and the same amount of MT were constructed using methods similar to those used to prepare the abovementioned hydrogel; the resulting hydrogels were named 5GelMA-DOPA@MT, 10GelMA-DOPA@MT, and 20GelMA-DOPA@MT.

#### 2.2.2. In Vitro Drug Release of Composite Hydrogel

The drug release behavior of different composite hydrogels was explored to determine the MT release curve. All of the drug-loaded composite hydrogels were immersed in PBS at 37°C and shaken at 100 rpm to simulate vitro release. The release medium was replaced by the same volume of fresh PBS at the set time points, and the samples were investigated by high-performance liquid chromatography (HPLC). The analysis was performed with an ODS column (100-5 C18 column, 250 × 4.6 mm, 5.0 *μ*m particle size, J&K Chemical, Ltd., Shanghai) at 30°C. To elute MT, the mobile phase consisted of deionized water and methanol (30 : 70*v*/*v*). The flow rate was 1.0 mL/min, and the detection wavelength was 277 nm.

#### 2.2.3. Water Absorption Experiment

To determine the ability of GelMA-DOPA at different concentrations and different types of hydrogels to absorb water, prepared hydrogel was freeze-dried to obtain lyophilized samples [40]. Subsequently, these samples were weighed (Wo) and immersed in PBS. At each set time point, all of the hydrogels were weighed (Wt); then, a curve to describe the ability of the hydrogel to absorb water was drawn. The percentage of water absorption was calculated by the following formula:

(1) $$\begin{array}{c} \text{Water absorption }\left(\%\right)=\left(\text{Wt}-\text{Wo}\right)/\text{Wo}\times 100. \end{array}$$

#### 2.2.4. In Vitro Degradation Tests

Degradation tests were performed according to previously reported procedures. Briefly, prepared hydrogel was immersed in PBS and incubated at 37°C for 24 h to achieve an equilibrium swelling state. Then, the swollen hydrogel was weighed, and its initial mass was recorded as w0. Swollen hydrogel was moved to and incubated in a solution of type II collagenase (2 U/mL) in PBS at 37°C while shaking at 100 rpm. At the set time point, the remaining hydrogel was weighed, and the mass was recorded as wt and used to describe the extent of degradation.

### 2.3. Mechanical Characterization

#### 2.3.1. Compression Experiment

Cylindrical crosslinked hydrogel was soaked in PBS at 37°C and incubated for 24 h until the hydrogel was swollen. Different concentrations of GelMA-DOPA ranging from 5 to 20% (*w*/*v*) were compressed at a speed of 0.5 mm/min using an equivalence force test instrument. GelMA, GelMA-COOH, GelMA-DOPA, and GelMA-DOPA@MT at 20% were also investigated with a similar method. All slopes in the linear region were utilized to determine the compressive moduli of various hydrogel samples.

#### 2.3.2. Adhesive Characterization of GelMA-DOPA

The adhesive property of GelMA-DOPA was explored by standard lap shear tests with some modifications. Briefly, 200 *μ*L of a precrosslinked solution of GelMA, GelMA-COOH, GelMA-DOPA, or GelMA-DOPA@MT (all of these precrosslinked solutions were at a concentration of 20%) was applied to the surface of the bone. The Ti side of a second bone was put into contact with the precrosslinked liquid, resulting in an overlapping (adhesive-bonded) area of approximately 1.0 × 2.0 cm^2^. After 10 seconds of UV irradiation, the samples were soaked in PBS for 2 h to create wet conditions. Then, a lap shear test was immediately performed under wet conditions until failure with a 50 N load cell at a speed of 1 mm/min.

### 2.4. In Vitro Biocompatibility of GelMA-DOPA

To examine the biocompatibility of GelMA-DOPA and its derivative, a cell counting kit-8 (CCK-8) assay was used to quantitatively assess biocompatibility, and a live/dead assay was used to qualitatively assess biocompatibility according to the product's instructions. MC3T3-E1 cells (2000 cells/well) were seeded in a 96-well plate and incubated with the leachates of GelMA, GelMA-DOPA, and GelMA-DOPA@MT. The level of dehydrogenase was measured by CCK-8 assay to indirectly determine the number of live cells at 1-day intervals. Fluorescence microscopy was utilized to observe the images from a live/dead assay at 488 and 568 nm excitation wavelengths. In addition, the cellular adhesion of the hydrogels was investigated by seeding MC3T3-E1 cells (1 × 10^4^ cells/well) on the surface of the abovementioned hydrogels in a 48-well plate. Then, the surfaces of the samples were observed by scanning electron microscopy (SEM) at 1-day intervals.

### 2.5. Cell Culture

MC3T3-E1 cells were cultured in mineralizing medium (*α*-minimum essential medium (*α*-MEM) (HyClone, Logan, UT, USA) containing 10% fetal bovine serum and 1% penicillin-streptomycin) in 5% CO_2_ at 37°C. To induce the differentiation of MC3T3-E1 cells, the cells were cultured in osteogenic differentiation medium (Dulbecco's modified Eagle medium containing 10% bovine serum, 100 *μ*g/mL streptomycin, 100 U/mL penicillin, 50 *μ*g/mL L-ascorbic acid, 100 nM dexamethasone, and 10 mM *β*-glycerol phosphate). Hydrogen peroxide (H_2_O_2_) and MT were purchased from Sigma (St. Louis, MO, USA). 3-TYP (a sirtuin 3 inhibitor) was purchased from Selleck. First, the cells were classified into four experimental groups: the (1) control group, cells treated with *α*-MEM; the (2) H_2_O_2_ group, cells treated with *α*-MEM containing H_2_O_2_ (400 *μ*M) once; the (3) MT group, cells treated with *α*-MEM containing H_2_O_2_ (400 *μ*M) and melatonin (10 *μ*M, dose-simulated melatonin systemic route to local system), and the (4) GelMA-DOPA@MT group, cells treated with *α*-MEM containing H_2_O_2_ (400 *μ*M) once and supplemented with GelMA-DOPA@MT (previously collected 20GelMA-DOPA@MT leachate). The media and melatonin in which the first three groups were grown was changed only every day, and medium containing leachate in which the last group was grown was collected daily. Then, the cells were subsequently classified into five experimental groups: the (1) control group, cells treated with *α*-MEM for 24 h, the (2) H_2_O_2_ group, cells treated with *α*-MEM for 12 h and H_2_O_2_ (400 *μ*M) for 12 h, the (3) GelMA-DOPA@MT group, cells pretreated with *α*-MEM for 12 h and then treated with *α*-MEM containing H_2_O_2_ (400 *μ*M) and GelMA-DOPA@MT (previously collected 20GelMA-DOPA@MT leachate) for 12 h, the (4) 3-TYP group, cells pretreated with 3-TYP (50 *μ*M) for 12 h and then treated with *α*-MEM supplemented with H_2_O_2_ (400 *μ*M) for 12 h, and the (5) GelMA-DOPA@MT+3-TYP group, cells pretreated with 3-TYP (50 *μ*M) for 12 h, followed by treatment with *α*-MEM supplemented with H_2_O_2_ (400 *μ*M) and GelMA-DOPA@MT (previously collected 20GelMA-DOPA@MT leachate) for 12 h.

### 2.6. Real-Time RT-PCR Analysis

We collected total RNA with TRIzol reagent (Invitrogen). The total RNA concentration was determined by a NanoDrop One (Thermo Fisher, USA), and RNA was used for reverse transcription in a 20 *μ*L reaction volume including PrimeScript RT Master Mix (Takara, Japan) and PCR amplification in a 20 *μ*L reaction volume including 10 *μ*L of Forget-Me-Not qPCR Master Mix (Biotium, USA), 0.5 *μ*L of each primer, 2 *μ*L of cDNA, and 7 *μ*L of RNase-free dH_2_O. The reaction was carried out with a LightCycler 96 real-time PCR detection system (Roche, USA). The cycle threshold values were normalized to the level of GAPDH. The following primer sequences were used to amplify ALP, OCN, Osterix, Bax, Bcl-2, Sirt3, and GAPDH: ALP forward 5′-CAGCGGGTAGGAAGCAGTTTC-3′ and reverse 5′-CCCTGCACCTCATCCCTGA-3′, OCN forward 5′-GAGGCTCTGAGAAGCATAAA-3′ and reverse 5′-AGGGCAATAAGGTAGTGAA-3′, Osterix forward 5′-TGAGCTGGAACGTCACGTGC-3′ and reverse 5′-AAGAGGAGGCCAGCCAGACA-3′, Bax forward 5′-AGACAGGGGCCTTTTTGCTA-3′ and reverse 5′-AATTCGCCGGAGACACTC-3′, Bcl-2 forward 5′-GCTACCGTCGTGACTTCGC-3′ and reverse 5′-CCCCACCGAACTCAAAGAAGG-3′, Sirt3 forward 5′-GAGCGGCCTCTACAGCAAC-3′ and reverse 5′-GGAAGTAGTGAGTGACATTGG-3′, and GAPDH forward 5′-GGTGAAGGTCGGTGTGAACG-3′ and reverse 5′-CTCGCTCCTGGAAGATGGTG-3′. Each sample was tested three times to reduce mistakes.

### 2.7. Protein Isolation and Western Blot Analysis

Protein samples were resolved by 15% sodium dodecyl sulfate polyacrylamide gel electrophoresis for 2 h and electrophoretically transmitted to a PVDF membrane (Merck Millipore, USA). After blocking nonspecific binding sites with 5% skim milk for 60 min at room temperature, the membranes were incubated overnight at 4°C with primary antibodies against SIRT3 (1 : 1000; ab189860), Bax (1 : 1000; ab32503), Bcl-2 (1 : 1000; ab59348), ALP (1 : 1000; ab95462), Osterix (1 : 500; ab22552), OCN (1 : 500; ab93876), *β*-actin (1 : 1000; ab8227), SOD2 (1 : 5000; ab13533), and Ac-SOD2 (1 : 5000; ab137037) (all from Abcam, Cambridge, UK). Then, the membranes were rinsed in Tris-buffered saline with Tween 20 and incubated with the corresponding secondary horseradish peroxidase-conjugated antibodies (1 : 1000) for 2 h at room temperature. The proteins were detected using chemiluminescent HRP substrates (Millipore Corporation, Billerica, MA, USA).

### 2.8. ALP Staining

After two weeks of culture in the osteogenic medium, MC3T3-E1 cells were incubated with alkaline phosphatase (ALP) stain. Briefly, we washed MC3T3-E1 cells three times with PBS. After fixation in 4% paraformaldehyde for 15 min, the cells were washed three times with PBS and then incubated in a BCIP/NBT working solution (Beyotime Biotech, Jiangsu, China) in the dark for 20 min. The staining outcomes were observed under a microscope.

### 2.9. Alizarin Red S Staining

After three weeks of culture in the osteogenic medium, MC3T3-E1 cells were stained with Alizarin red S (ARS). Briefly, we washed MC3T3-E1 cells three times with PBS, followed by fixation in 4% paraformaldehyde for 20 min at 4°C. Then, the cells were rinsed and incubated in an ARS staining solution (pH 4.2; Cyagen Biosciences, Santa Clara, CA, USA) for 20 min. Finally, ddH_2_O was used to wash the cells three times.

### 2.10. TUNEL Staining

After three days of culture in osteogenic medium, MC3T3-E1 cells were examined by a One Step TUNEL Apoptosis Assay Kit (Beyotime Biotech, Jiangsu, China), and the cells were observed under fluorescence microscopy to assess apoptosis.

### 2.11. Animals

Female Sprague-Dawley (SD) rats (8 weeks old, 250 ± 20 g) were purchased from JOINN Laboratories (Suzhou, China). All of the cells in which the animals were fed were specific pathogen-free- (SPF-) class isolators. Experiments involving animals were approved by the Institute of Animal Care Committee of Zhangjiagang Traditional Chinese Medicine Hospital.

### 2.12. Surgical Procedures

All of the animals received anesthesia. Bilateral ovariectomy (OVX) was performed in 24 rats, and sham operations were performed in the remaining 6 rats. After 3 months, the bone mineral density (BMD) of all rats was measured using dual-energy X-ray absorptiometry (DEXA; Lunar Corporation, Madison, WI, U.S.) to assess whether the osteoporosis model had been successfully developed. Once success was determined, 30 rats underwent bilateral distal femoral titanium screw implantation with sterilized titanium screws (Adil, Suzhou, China). The implants were placed laterally into the distal femur of each rat. Depending on the group, no specific drugs were injected around the implant. Then, the 30 animals were randomly classified into five groups: implant only (control group), ovariectomy+implant (OVX group), OVX+implant+GelMA (GelMA group), OVX+implant+MT (MT group), and OVX+implant+GelMA-DOPA@MT (GelMA-DOPA@MT group) with each group including 6 animals. The MT and GelMA-DOPA@MT groups received the daily i.p. administration of MT (Sigma) at 50 mg/kg of body weight between 5 : 00 pm and 6 : 00 pm for 4 weeks following implant installation, while rats in the other groups were injected with normal saline under the same conditions. In addition, all of the rats were injected i.p. 10 mg/kg calcein (Sigma) 10 and 2 days before euthanasia. In the last part of the experiment, the rats were sacrificed, followed by the collection of their bilateral femurs for subsequent studies.

### 2.13. Radiological Analyses

Femurs containing the titanium implants (*n* = 6 in each group) were measured with high-resolution microcomputed tomography (*μ*CT, SkyScan 1176; SkyScan, Knotich, Belgium). The femurs were scanned with 18 *μ*m per layer, and the X-ray parameters were set at a voltage of 50 kV, a current of 500 *μ*A, and a 0.7° rotational step. A round region with a diameter of 1.8 mm around one-third along the titanium rods of distal femurs was chosen to evaluate related morphometric parameters, including BMD (g/cm^3^), bone volume/total volume (BV/TV, %), bone surface/bone volume (BS/BV, 1/mm), bone surface/total volume (BS/TV, 1/mm), and trabecular number (TbN, 1/mm). Relevant three-dimensional (3D) images were analyzed after processing.

### 2.14. Histological and Immunohistochemical Analyses

All of the femurs were preserved at 4°C for histological and immunohistochemical analyses after *μ*CT. Each entire left femur (*n* = 6 per group) was used for hard tissue section staining. After embedded with epoxy resin (45347, Sigma), each specimen was cut into 10 *μ*m thick sections along the vertical axis with a hard tissue slicer (EXAKT 300CP, Germany). The obtained hard tissue sections were used for calcein staining, whose images determined by fluorescence microscopy. And the mineral apposition rate (MAR) was calculated using Bioquant Osteo 2017 (BIOQUANT Image Analysis Corporation, USA).

The complete femurs were decalcified for 1 month with 15% ethylenediaminetetraacetic acid (EDTA, Sigma), and the specimens (right rat femurs, *n* = 6 per group) were embedded in paraffin after the Ti screws had been removed. Cutting the specimens into 5 *μ*m sections which used for hematoxylin and eosin (H&E) staining. Finally, sealed the sections by means of neutral balsam (Solarbio) and photographed. With optical microscopy, the BV/TV and inflammatory area were calculated using Bioquant Osteo 2017.

For immunofluorescence staining and terminal deoxynucleotidyl transferase dUTP nick end labeling (TUNEL) staining (Beyotime Biotech, China). The selected region of interest (ROI) was around the Ti screws below the epiphysis. In brief, the sections were dewaxed with xylene and then subjected to gradient hydration and antigen retrieval with hyaluronidase for 1 h at 37°C and pepsin for 25 min at room temperature. The sections were then blocked with serum for 30 min. Next, the paraffin sections were incubated with primary antibodies ALP (ab95462) and Rux2 (ab192256) (all from Abcam) for 12 h at 4°C. Subsequently, the sections were rinsed with PBS and incubated with secondary antibodies (ab150077) for 60 min at room temperature in dark. Then, the sections were rinsed with PBS and incubated with TUNEL test solution for 30 min at 37°C. Afterwards, rinsed with PBS and added mounting medium with DAPI (ab104139). Finally, the slides were stored in the dark at 2-8°C for 10 min and determined by fluorescence microscopy. Positive staining was determined by Bioquant Osteo 2017. The selected region of interest (ROI) was around the Ti screws below the epiphysis.

### 2.15. Statistical Analysis

Results are represented as the means ± standard deviations. The Kolmogorov-Smirnov test was employed to test statistical normality. Statistical significance was determined by one-way ANOVA with the Tukey's multiple comparisons test employing SPSS 25.0, with *p* < 0.05 indicating a statistically significant difference.

## 3. Results

### 3.1. In Vitro Characterization of GelMA-DOPA@MT

**Figure 1:**
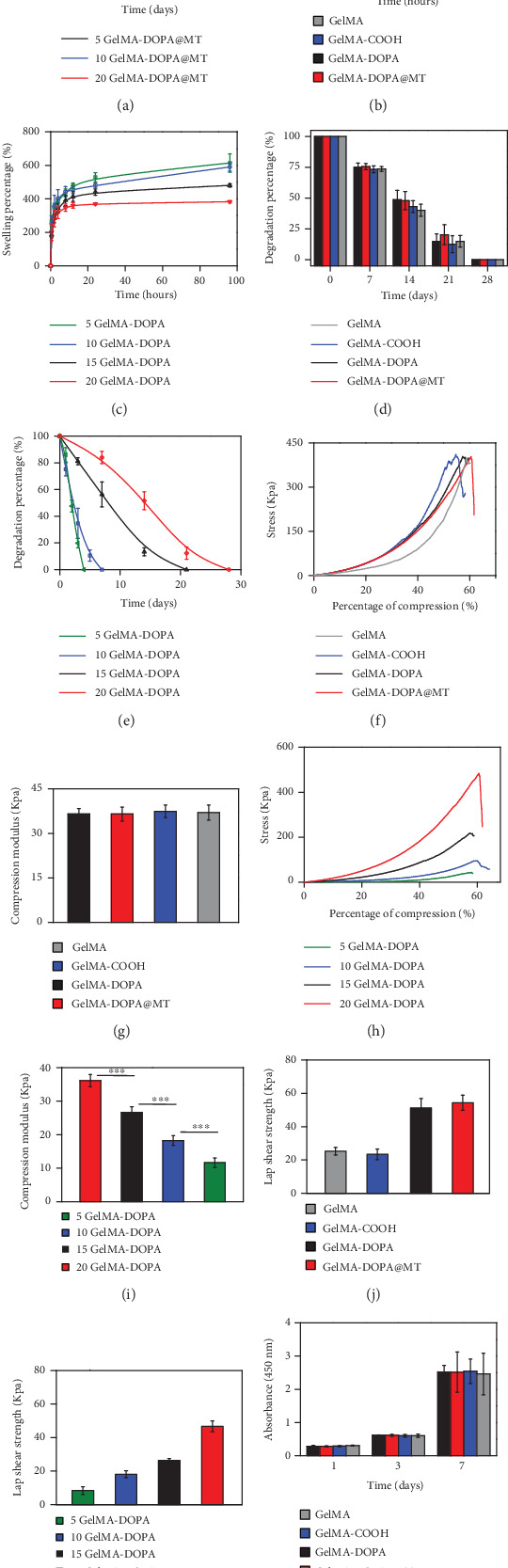
In vitro characterization of GelMA-DOPA@MT. (a) In vitro drug release behavior. (b) Swelling of GelMA with different modifications. (c) GelMA-DOPA at different concentrations. (d) Degradation of GelMA with different modifications. (e) GelMA-DOPA at different concentrations. (f, g) Compressive testing of GelMA with different modifications and Young's modulus. (h, i) Compressive testing on GelMA-DOPA at different concentrations and Young's modulus. (j, k) Investigation of the adhesive properties of GelMA-DOPA at different concentrations and Young's modulus. (l) Cytotoxicity of GelMA derivatives. (*n* = 3 per group, ∗*p* < 0.05, ∗∗*p* < 0.01, ∗∗∗*p* < 0.001).

The drug release behaviors of GelMA-DOPA with different amounts of MT liposomes were investigated in vitro. As shown in Figure 1(a), the samples exhibited various release characteristics because of differences in their hydrogel network. Among the samples, 5GelMA-DOPA@MT showed the shortest sustained release period of approximately 5 days, and 20GelMA-DOPA@MT exhibited the longest continuous release time of almost 25 days. Differences in the release characteristics between the samples can be attributed to the density of the hydrogel network. For example, 5GelMA-DOPA@MT had the loosest hydrogel network, while 20GelMA-DOPA@MT exhibited the densest hydrogel construction. 20GelMA-DOPA@MT released an inconspicuous burst of MT in the first few days and continuously released MT for approximately 20 days, which laid the groundwork to promote bone regeneration.

As they are important characteristics, the water absorption and degradation of the hydrogels were explored. As shown in Figure 1(b), when hydrogels were prepared at the same concentration (20%), there was no dramatic difference in water absorption among hydrogels composed of different materials (GelMA, GelMA-COOH, GelMA-DOPA, and GelMA-DOPA@MT). As shown in Figure 1(c), there were significant differences in water absorption among hydrogels at different concentrations (5-20%), and water absorption ranged from 614.2 ± 54.9% for 5GelMA-DOPA to 382.8 ± 6.2 for 20GelMA-DOPA. However, degradation of the hydrogels exhibited a tendency similar to that of water absorption, as shown in Figures 1(d) and 1(e). Hydrogels at the same concentration (20%) degraded at similar rates, indicating that GelMA, GelMA-COOH, GelMA-DOPA, and GelMA-DOPA@MT would be completely degraded after 27 days of soaking. Meanwhile, hydrogels at various concentrations degraded for different lengths of time ranging from 5 days for 5GelMA-DOPA to 28 days for 20GelMA-DOPA.

In implantation, the ability to bear certain external force is crucial; thus, we further characterized GelMA hydrogels with different modifications by compression testing. As shown in Figures 1(f) and 1(g), there were no notable differences in the compression moduli of hydrogels at the same concentration, which were 36.5 ± 1.9 kPa for GelMA, 37.4 ± 2.1 kPa for GelMA-DOPA, and 37.0 ± 2.5 kPa for GelMA-DOPA@MT. Drug-loaded composite hydrogels exhibited mechanical properties similar to those of the other three groups, providing an excellent foundation for multifunctional implants. Moreover, according to the results shown in Figures 1(h) and 1(i), the compression test indicated the expected positive correlation between GelMA-DOPA concentrations and the compressive moduli of the resulting hydrogels, which ranged from 11.6 ± 1.5 kPa for 5GelMA-DOPA hydrogels to 36.1 ± 1.8 kPa for 20GelMA-DOPA hydrogels.

Considering the working conditions of implants, improving adhesion to the surfaces of bones and Ti in wet environments is critical to the success of a multifunctional hydrogel. Therefore, lap shear tests were performed to characterize the in vitro adhesive properties of various kinds of GelMA-DOPA under a wet environment. As shown in Figures 1(j) and 1(k), GelMA and GEL-COOH showed limited adhesive properties, but GelMA-DOPA and GelMA-DOPA@MT exhibited significant adhesive properties. The lap shear strength, which ranged from 9.6 ± 1.9 kPa for 5GelMA-DOPA hydrogels to 46.7 ± 4.9 kPa for 20GelMA-DOPA hydrogels, increased with increasing GelMA-DOPA concentration. This indicates that increasing prepolymer concentrations with an increased DOPA content improves adhesion.

**Figure 2:**
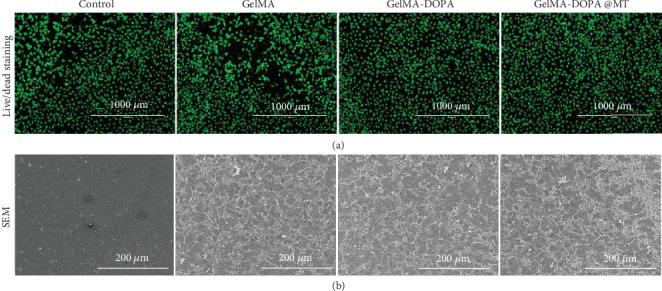
In vitro biocompatibility of GelMA-DOPA@MT. (a) Results of live/dead staining of MC3T3 cells cultured on the surfaces of GelMA derivatives. Scale bar: 1000 *μ*m. (b) SEM imaging of MC3T3 cells cultured on the surfaces of GelMA derivatives. Scale bar: 200 *μ*m.

In order to quantify cell proliferation, CCK-8 assays were carried out at 1-day intervals, as shown in Figure 1(l) and Table S1. The optical density (OD) among the four groups was not significantly different and ranged from 0.3 after 1 day to 2.5 after 7 days. Furthermore, cell viability was observed by live/dead staining after 7 days of incubation. As shown in Figure 2(a), the cells in all groups exhibited high viability and excellent dispersion, which shows that GelMA-DOPA@MT exhibits suitable cytocompatibility. In addition, MC3T3-E1 cells were seeded on the surface of different hydrogels, and their cellular morphology and adherence were monitored. The SEM images shown in Figure 2(b) reveal that cells grown on the surface of GelMA-DOPA@MT had a morphology similar to that of cells grown on the GelMA surface at the same time points, indicating that the presence of MT and drug release did not affect cell adherence.

All of the abovementioned results suggest that the ability of GelMA-DOPA@MT to promote MC3T3-E1 cell adhesion and proliferation was not significantly different from that observed in the GelMA, GelMA-DOPA, and control groups, establishing the foundation for further research.

### 3.2. GelMa-DOPA@MT Promotes the Differentiation of Osteoblasts and Inhibits Their Apoptosis

**Figure 3:**
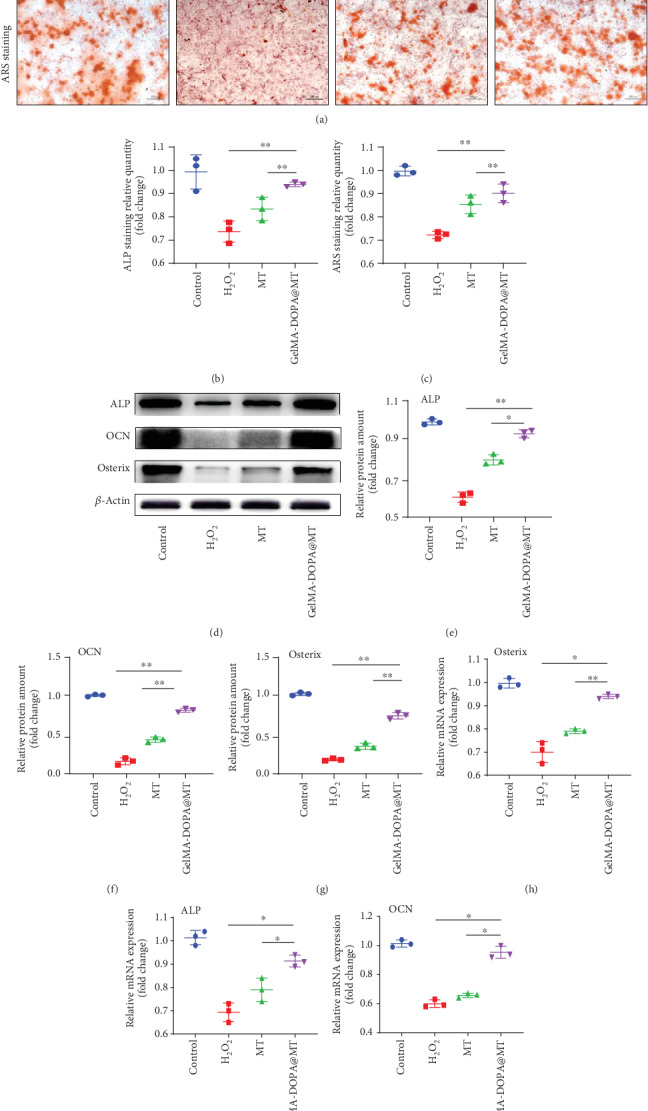
GelMA-DOPA@MT promotes the differentiation of osteoblasts in vitro. (a) Representative images showing ARS and ALP staining, with red and black arrows indicating calcium nodules. Scale bar: 50 *μ*m. (b, c) Quantitative analysis of ARS and ALP staining. *n* = 3 per group, ∗*p* < 0.05, ∗∗*p* < 0.01, vs. the GelMA-DOPA@MT group. (d) Cell lysate was subjected to Western blotting with antibodies against the osteogenesis-specific proteins ALP, OCN, and Osterix. (e–g) Quantification of ALP, OCN, and Osterix protein levels, *n* = 3 per group, ∗*p* < 0.05, ∗∗*p* < 0.01, vs. the GelMA-DOPA@MT group. (h, i) qPCR analysis of the expression of the osteogenic genes ALP, OCN, and Osterix in the experimental group, *n* = 3 per group, ∗*p* < 0.05, ∗∗*p* < 0.01, vs. the GelMA-DOPA@MT group.

As shown in Figure 3(a), after MC3T3-E1 cells in different groups underwent treatment, ALP staining showed that the number of positive cells decreased significantly after H_2_O_2_ treatment compared with that in the control group. However, drug intervention reversed this phenomenon. Quantitative analysis indicated more positive cells in the GelMA-DOPA@MT group than in the MT group. Similar results were also observed by ARS staining. The number of calcium nodules in the H_2_O_2_ group was extremely small but significantly increased by drug intervention, especially that in the GelMA-DOPA@MT group. Quantitative analysis also indicated that the GelMA-DOPA@MT group exhibited increased osteogenesis (by 1.2-fold vs. the MT group).

To further observe the ability of GelMA-DOPA@MT to induce osteogenic differentiation, RT-PCR showed that after induction by H_2_O_2_, the mRNA levels of Osterix (0.7-fold, Figure 3(h)), ALP (0.69-fold, Figure 3(i)), and OCN (0.6-fold, Figure 3(j)) were decreased compared with those in the control group. However, drug intervention reversed this phenomenon. As expected, GelMA-DOPA@MT obviously increased gene expression compared with that in the MT group (ALP, 1.1-fold; OCN, 1.4-fold; and Osterix, 1.2-fold). Western blotting showed increased protein levels of ALP, OCN, and Osterix in the GelMA-DOPA@MT group even in the presence of H_2_O_2_ (Figures 3(d)–3(g)). In addition, similar results were observed in rat BMSCs, and GelMA-DOPA@MT significantly attenuated H_2_O_2_-induced inhibition on osteoblastic differentiation (Figure S1).

**Figure 4:**
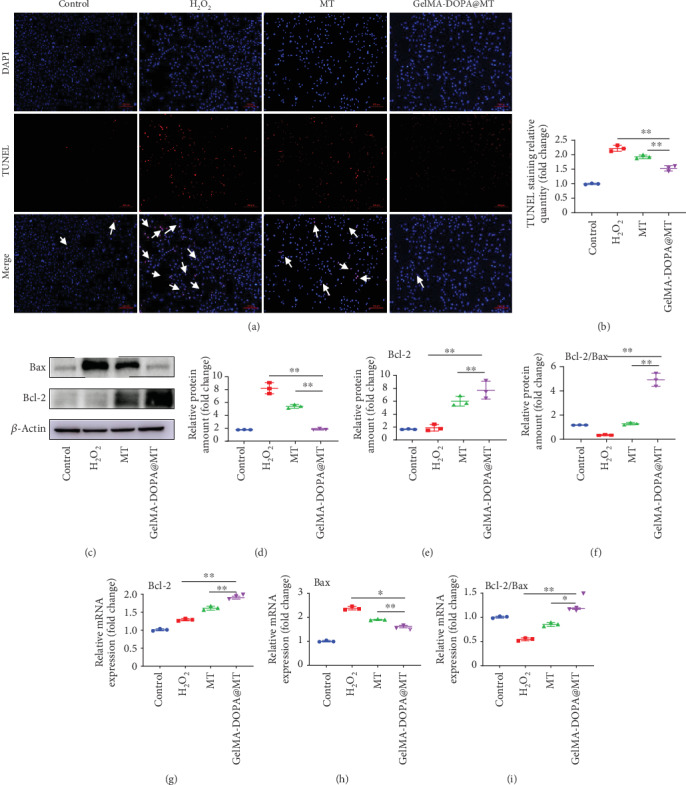
GelMA-DOPA@MT inhibits the apoptosis of osteoblasts in vitro. (a) Representative images showing TUNEL staining, with yellow arrows indicating apoptotic cells. Scale bar: 50 *μ*m. (b) Quantitative analysis of TUNEL staining results. *n* = 3 per group, ∗*p* < 0.05, ∗∗*p* < 0.01, vs. the GelMA-DOPA@MT group. (c) Cell lysate was subjected to Western blotting with antibodies against the apoptosis-specific proteins Bax and Bcl-2. (d–f) Quantification of Bax and Bcl-2 protein levels, *n* = 3 per group, ∗*p* < 0.05, ∗∗*p* < 0.01, vs. the GelMA-DOPA@MT group. (g–i) qPCR analysis of the expression of the apoptosis-specific genes Bcl-2 and Bax in the experimental group. *n* = 3 per group, ∗*p* < 0.05, ∗∗*p* < 0.01, vs. the GelMA-DOPA@MT group.

Oxidative stress caused by H_2_O_2_ is often accompanied by cell apoptosis. We observed by TUNEL staining that H_2_O_2_ significantly increased apoptosis in MC3T3-E1 cells (Figure 4(a)), while the apoptosis of MC3T3-E1 cells in the MT and GelMA-DOPA@MT groups was inhibited. Quantitative analysis (Figure 4(b)) showed that apoptosis in MC3T3-E1 cells in the GelMA-DOPA@MT group (0.8-fold) was decreased compared to that in MC3T3-E1 cells in the MT group. Further, Western blotting (Figures 4(c)–4(f)) also showed that H_2_O_2_ significantly increased the protein level of Bax and lowered the ratio of Bcl-2/Bax compared with those in the control group. Conversely, drug intervention reduced the Bax protein level and increased the ratio of Bcl-2/Bax, and the effects of drug intervention in the GelMA-DOPA@MT group were more obvious than those in the MT group. RT-PCR also showed similar results (Figures 4(g)–4(i)).

### 3.3. Effects of GelMA-DOPA@MT on the SIRT3/SOD2 Signaling Pathway

**Figure 5:**
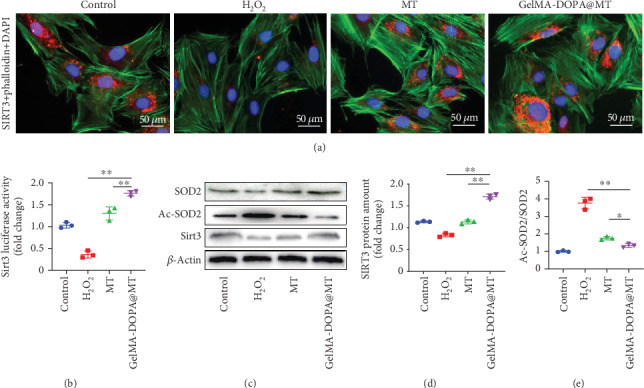
Effects of GelMA-DOPA@MT on the SIRT3/SOD2 signaling pathway. (a, b) Immunofluorescent staining for SIRT3 and quantitative analysis. Scale bar: 50 *μ*m. *n* = 3 per group, ∗*p* < 0.05, ∗∗*p* < 0.01, vs. the GelMA-DOPA@MT group. (c) Western blotting of cell lysates with antibody against SIRT3, SOD2, and Ac-SOD2. (d) Quantitative analysis of SIRT3 protein levels and (e) the Ac-SOD2/SOD2 ratio in the experimental group, *n* = 3 per group, ∗*p* < 0.05, ∗∗*p* < 0.01, vs. the GelMA-DOPA@MT group.

Previous research has shown that MT inhibits mitochondrial oxidative stress via the SIRT3/SOD2 signaling pathway. Because mitochondrial oxidative stress is an important factor in apoptosis, we focused on changes in SIRT3 in a mechanistic study. We first demonstrated the expression of SIRT3 in MC3T3-E1 cells by immunohistochemistry (IHC). As shown in Figures 5(a) and 5(b), H_2_O_2_ significantly reduced the expression of SIRT3, while drug intervention increased its expression, and SIRT3 activity in the GelMA-DOPA@MT group was significantly higher than that in the MT group. We further evaluated the protein and gene expression levels of SIRT3 and SOD2 (Figures 5(c)–5(e)). As expected, H_2_O_2_ obviously decreased the protein level of SIRT3 and increased the ratio of Ac-SOD2/SOD2 compared to those in the control group. Conversely, drug intervention reversed this phenomenon. Meanwhile, the effect of MT on the GelMA-DOPA@MT group was more significant than that on the MT group.

**Figure 6:**
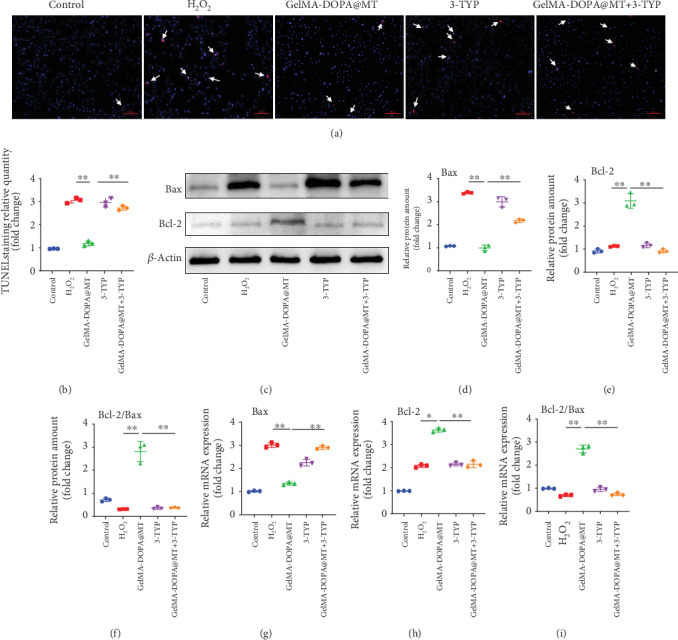
Melatonin inhibits osteoblast apoptosis by activating the sirt3 pathway and promotes osteogenic differentiation. (a, b) TUNEL staining and quantitative analysis, with yellow arrows indicating apoptotic cells. *n* = 3 per group, ∗*p* < 0.05, ∗∗*p* < 0.01, vs. the GelMA-DOPA@MT group. (c) Cell lysate was subjected to Western blotting with antibodies against the apoptosis-specific proteins Bax and Bcl-2. (d–f) Quantitative analysis of Bax and Bcl-2 protein levels, *n* = 3 per group, ∗*p* < 0.05, ∗∗*p* < 0.01, vs. the GelMA-DOPA@MT group. (g–i) qPCR analysis of the expression of the apoptosis-specific genes Bax and Bcl-2 in the experimental group, *n* = 3 per group, ∗*p* < 0.05, ∗∗*p* < 0.01, vs. the GelMA-DOPA@MT group.

**Figure 7:**
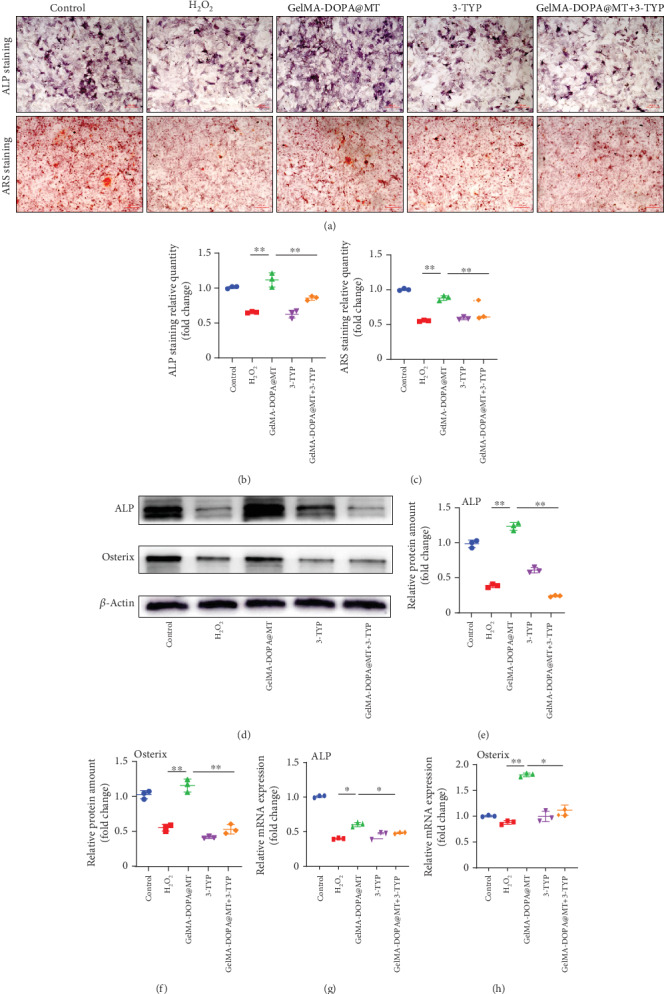
Melatonin promotes osteogenic differentiation by activating the sirt3 pathway. (a) Representative images showing cellular ARS and ALP staining. (b, c) Quantitative analysis of the results of ARS and ALP staining. *n* = 3 per group, ∗*p* < 0.05, ∗∗*p* < 0.01, vs. the GelMA-DOPA@MT group. (d) Cell lysate was subjected to Western blotting with antibodies against the osteogenesis-specific proteins ALP and Osterix. (e, f) Quantitative analysis of ALP and Osterix protein levels, *n* = 3 per group, ∗*p* < 0.05, ∗∗*p* < 0.01, vs. the GelMA-DOPA@MT group. (g, h) qPCR analysis of the expression of the osteogenesis-specific genes ALP and Osterix in the experimental group, *n* = 3 per group, ∗*p* < 0.05, ∗∗*p* < 0.01, vs. the GelMA-DOPA@MT group.

A SIRT3-selective inhibitor (3-TYP) was used in the next experiment. TUNEL staining showed that the inhibitory effect of MT on apoptosis was inhibited by 3-TYP, and quantitative analysis showed that GelMA-DOPA@MT+3-TYP treatment did not effectively prevent the apoptosis of MC3T3-E1 cells in the GelMA-DOPA@MT+3-TYP group (by 2.5-fold) compared to that in the MT group (Figures 6(a) and 6(b)). Meanwhile, RT-PCR and Western blotting (Figures 6(c)–6(f)) confirmed that 3-TYP reversed the effective antiapoptotic effect of GelMA-DOPA@MT seen in the GelMA-DOPA@MT treatment group (RT: Bax, 2.1-fold; Bcl-2, 0.6-fold) (Figures 6(g) and 6(i)). Finally, ALP and ARS staining showed that 3-TYP significantly inhibited ALP expression (by 0.5-fold vs. ALP expression in the GelMA-DOPA@MT group) and the formation of calcium nodules (by 0.4-fold vs. calcium nodule formation in the GelMA-DOPA@MT group) (Figures 7(a)–7(c)). RT-PCR and Western blotting further demonstrated that 3-TYP increased the expression of ALP and Osterix (RT: ALP, 0.6-fold; Osterix, 0.6-fold) (Figures 7(d)–7(h)).

### 3.4. GelMA-DOPA@MT Promotes Bone Mass around Ti Implants

**Figure 8:**
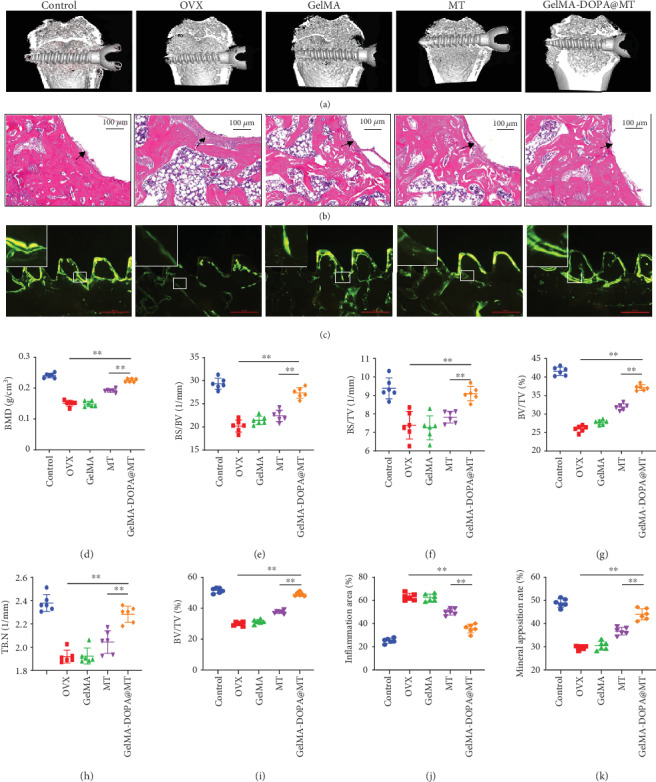
GelMA-DOPA@MT prevents ovariectomy- (OVX-) induced bone loss in vivo. (a) Representative 3D reconstructions of *μ*CT images and (d) BMD within the ROI (region of interest) were calculated. (e) BS/BV, (f) BS/TV, (g) BV/TV, and (h) TbN. (b) Representative paraffinized section following H&E staining. Black arrows represent inflammatory fibrous tissue, Scale bar: 100 *μ*m. (c) Representative histological images around the screw showing calcein levels. Scale bar: 500 *μ*m. (i) BV/TV and (j) inflammatory area. (k) Mineral apposition rate. *n* = 6 per group, ∗*p* < 0.05, ∗∗*p* < 0.01, vs. the GelMA-DOPA@MT group.

Trabecular microstructures around the implanted Ti screws were also evaluated by 3D *μ*CT analysis (Figure 8(a)). Compared with that in the control group, the bone mass in the OVX group was significantly reduced, as shown by the following *μ*CT data: BMD (0.241 ± 0.007 vs. 0.151 ± 0.009, respectively, g/cm^3^), BV/TV (41.56 ± 1.14 vs. 26.03 ± 0.86, respectively, %), BS/BV (29.36 ± 1.22 vs. 20.22 ± 1.29, respectively, 1/mm), BS/TV (9.38 ± 0.56 vs. 7.39 ± 0.73, respectively, 1/mm), and TbN (2.38 ± 0.07 vs. 1.92 ± 0.05, respectively, 1/mm). However, after MT or GelMA-DOPA@MT was injected around the Ti screw, the bone mass increased, and the bone mass in the GelMA-DOPA@MT group was significantly greater than that in the MT group, as shown by the following *μ*CT data: BMD (0.225 ± 0.006 vs. 0.192 ± 0.006, respectively, g/cm^3^), BV/TV (37.13 ± 0.78 vs. 31.93 ± 0.87, respectively, %), BS/BV (27.38 ± 1.14 vs. 22.42 ± 1.22, respectively, 1/mm), BS/TV (9.09 ± 0.39 vs. 7.82 ± 0.32, respectively, 1/mm), and TbN (2.28 ± 0.07 vs. 2.04 ± 0.10, respectively, 1/mm) (Figures 8(d)–8(h)). These results indicated the presence of more trabecular bone around Ti screws in the GelMA-DOPA@MT-treated group than in the MT-treated group. Both groups exhibited more trabecular bone than the OVX group. In summary, the degree of bone formation around Ti screws in vivo was efficiently promoted by GelMA-DOPA@MT injection. Consistent with the *μ*CT results, histological analysis further confirmed the bone-protective effects of MT in osteoporotic rats. H&E staining of the surrounding tissues clearly showed fibrous capsule between the bone matrix and the Ti rod in the OVX- and GelMA-treated groups, indicating a significant inflammatory reaction induced by oxidative stress. In contrast, a more complete bone structure was observed in the MT- and GelMA-DOPA@MT-treated groups than that in the control group (Figure 8(b)). Single and double fluorochrome labeling, a direct histologic marker of bone formation, was more apparent, and the distance between double labels was shorter in the OVX group than in the control group, whereas GelMA-DOPA@MT treatment significantly increased single and double label interlabel width compared with that in the MT group (Figure 8(c)).

**Figure 9:**
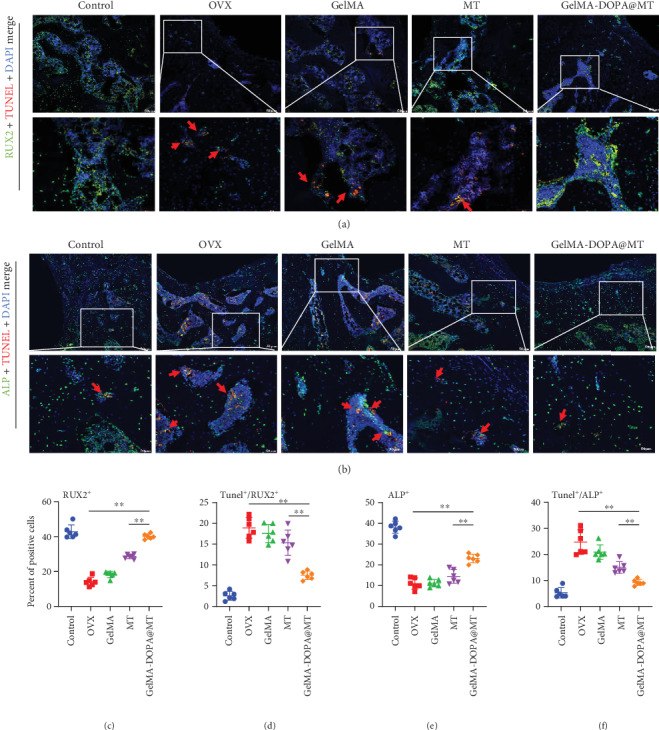
Fluorescence staining of tissue sections was used to analyze the effect of GelMA-DOPA@MT on osteoblast apoptosis around the implant. (a) Representative images showing cells stained for the osteogenic gene RUX2 (green), apoptosis (red), and nuclei (blue) observed by confocal microscopy. (b) Representative images showing cells stained for the osteogenic gene ALP (green), apoptosis (red), and nuclei (blue) observed by confocal microscopy. The red arrow indicates the apoptotic region during osteogenesis. Scale bar: 50 *μ*m. (c) Percent of positive cells of RNX2. (d) Ratio of Tunel/RUX2. (e) Percent of positive cells of ALP. (f) Ratio of Tunel/ALP. *n* = 6 per group, ∗*p* < 0.05, ∗∗*p* < 0.01, vs. the GelMA-DOPA@MT group.

To further confirm the inhibitory effect of GelMA-DOPA@MT on apoptosis in osteoblasts in vivo, further study of the histological sections was performed by immunofluorescence staining. As shown in Figures 9(a)–9(f) and observed under confocal microscopy, the positive area in the OVX group was significantly larger than that in the control group, while in the MT-treated group, the positive area was smaller than that in the OVX group, and the effect in the GelMA-DOPA@MT-treated group was more significant. These results confirmed that GelMA-DOPA@MT can effectively reduce the apoptosis of osteoblasts in vivo.

## 4. Discussion

Approximately 8.9 million fractures are caused by osteoporosis each year in the world. On average, one osteoporotic fracture occurs every 3 sec [41]. Osteoporosis will become a serious challenge for health authorities as many countries in the world have an aging society [2, 3, 5]. Surgical treatment after fracture usually uses internal fixation to restore bone integrity and restore mechanical support. Internal fixation stability is an important factor for postoperative recovery from fractures [42]. Surgical treatment after fracture usually uses internal fixation to restore bone integrity and restore mechanical support. Internal fixation stability is an important factor for postoperative recovery from fractures [8, 43]. Importantly, we demonstrate that the apoptosis of osteoblasts may exacerbate deterioration due to osteoporosis and ultimately lead to loosening of the implant. Unfortunately, in patients with osteoporosis, there is no good method to protect the bone around the implant. Bone reconstruction is a dynamic process in which the homeostasis between bone resorption and bone formation is critical [44]. Importantly, we demonstrate that the apoptosis of osteoblasts may exacerbate deterioration due to osteoporosis and ultimately lead to loosening of the implant. Unfortunately, in patients with osteoporosis, there is no good method to protect the bone around the implant. Bone reconstruction is a dynamic process in which the homeostasis between bone resorption and bone formation is critical [45, 46]. In osteoporosis, the increased production of ROS may contribute significantly to the loss of bone around the implant [47]. Recently, an increasing number of studies have shown that oxidative stress-induced osteoblast apoptosis plays an important role in osteoporosis. These findings suggest that inhibiting osteoblast apoptosis can reduce the impact of osteoporosis [48].

MT, which is synthesized from serotonin in the human pineal gland, is involved in the regulation of many physiological processes, such as sleep induction and anti-inflammatory, antitumor, and antioxidant functions [20]. In recent years, the protective effect of MT on mitochondrial oxidative stress has been reported [28, 29, 49]. We studied the ability of GelMA-DOPA@MT extract to promote osteoblasts and inhibit osteoblast apoptosis in vitro. MT inhibited mitochondrial oxidative stress through the SIRT3 signaling pathway and attenuated the apoptosis of MC3T3-E1 osteoblasts. Sirtuins (SIRTs), a family of nicotinamide adenine dinucleotide- (NAD+-)dependent deacetylases with seven members (SIRT1-SIRT7), among which SIRT1 and SIRT3 are the most extensively studied [50], have been shown to play an important role in apoptosis. SIRT3, a highly conserved NAD^+^-dependent deacetylase located in the mitochondria considered to be the most important acetyl lysine deacetylase, which regulates a variety of proteins to modulate mitochondrial effects and ROS production [51]. The role of mitochondrial oxidative stress has been widely confirmed. In our oxidative stress model, we observed a significant decrease in SIRT3 expression in MC3T3-E1 osteoblast precursor cells. MT increased SIRT3 expression and inhibited cell apoptosis compared to those in the H_2_O_2_ group. This effect of MT was significantly attenuated by a SIRT3-specific inhibitor. These results confirm the protective effect of MT on mitochondrial oxidative stress through the SIRT3 signaling pathway, reducing apoptosis-induced oxidative stress.

Studies in animals have shown that the systemic administration of MT enhances bone formation or integration [26, 52]. Orally and i.p. injected MT increased the volume of femoral cortical bones in mice. Similarly, Ostrowska et al. [53] found that i.p. injection of exogenous MT in rats following pineal gland resection inhibited biochemical markers of bone metabolism, such as ALP. Takechi et al. [54] reported that MT promoted osseointegration around tibial titanium implants in Wistar rats. Zhou et al. [55] showed that MT can accelerate bone formation around dental titanium implants. Therefore, the systemic administration of MT is a potential treatment for diseases such as osteoporosis that involve low bone density throughout the skeletal system. Similarly, studies by Calvoguirado et al. [56] have shown that the topical application of MT increases calcium deposition around implants. Furthermore, the cortical bone was regenerated around the width and length of tibial implants faster in rabbits that received topical MT than that in untreated rabbits. Therefore, topical MT administration induces bone healing without affecting other systems, which is often a powerful tool for treating local bone tissue.

However, the initial burst of MT release and short administration period are obvious shortcomings of the topical application of MT in bone repair and could limit the clinical application of MT to some extent. Therefore, it is especially necessary to establish a long-term drug delivery system for the local administration of MT. Liposomes have been used as FDA-approved drug carriers to control drug release for several decades [57]. Additionally, due to their bilayer structure, liposomes can effectively carry both hydrophilic and hydrophobic drugs [34, 35]. However, it is difficult to maintain a certain therapeutic drug concentration during liquid administration. GelMA-DOPA, a gelatin derivative, shows satisfactory adhesive properties, biodegradability, and biocompatibility [31–33]. Before its exposure to UV light, a precrosslinked GelMA solution could be injected and used to fill the gap between the implant and the cancellous bone [58]. After crosslinking, a complete amalgam between the implant and bone is generated. In this study, we prepared MT liposomes (MT@liposomes), which were then mixed with a GelMA-DOPA solution to prepare a precrosslinked solution. Then, the solution was injected into the bone defect area, followed by its exposure to UV light. Finally, a sustained MT release system with favorable adhesive properties to join implants and the surrounding tissues was generated.

Nevertheless, there were several limitations in this study. On the one hand, as we all know, bone metabolism includes both bone formation and bone resorption. However, our results only showed that GelMA-DOPA@MT increased bone mass around Ti screws through promotion of bone formation. Although Ping et al. [59] demonstrated that MT inhibits bone resorption at osteolytic sites caused by titanium particle-stimulation, whether GelMA-DOPA@MT can affect bone resorption around Ti screws requires further research. This question is currently the focus of ongoing study in our laboratory. On the other hand, the OVX-induced osteoporosis model applied in our study is different from osteoporosis with natural aging. Regardless of human or animals, age can affect the number, proliferation, and differentiation of cells [60, 61]. Therefore, the aging osteoporosis model which was more clinically relevant is necessary to verify the effect of GelMA-DOPA@MT on the stability of implants. Additionally, we need to use human-derived osteoblasts, osteoclasts, or human cell lines to further make it more clinically relevant in the following experiments.

## 5. Conclusion

In this study, we developed a novel MT sustained release system, GelMA-DOPA@MT, which can attach to the surface of Ti screws by photocrosslinked under UV light. In addition, GelMA-DOPA@MT can suppress osteoblast apoptosis induced by oxidative stress, thereby promoting osteogenic differentiation and improving bone quality around the prosthesis through activating the SIRT3/SOD2 signaling pathway. This system of local, sustained MT release is a suitable candidate for the treatment of implant loosening in patients with osteoporosis.

## Data Availability

The data used to support the findings of this study are available from the corresponding author upon request.

## Supplementary Materials

Supplementary materials Figure S1: GelMA-DOPA@MT promotes the differentiation of osteoblasts in vitro (BMSC). Table S1: the quantitative results of CCK8 staining.
