# Supplementary material for: Sustained Release of Melatonin from GelMA Liposomes Reduced Osteoblast Apoptosis and Improved Implant Osseointegration in Osteoporosis
Source: Oxid Med Cell Longev. 2020 May 28;2020:6797154. doi: 10.1155/2020/6797154 (PMC7275204; doi:10.1155/2020/6797154)
Supplement: Supplementary materials — Figure S1: GelMA-DOPA@MT promotes the differentiation of osteoblasts in vitro (BMSC). Table S1: the quantitative results of CCK8 staining. [file 6797154.f1.pdf]

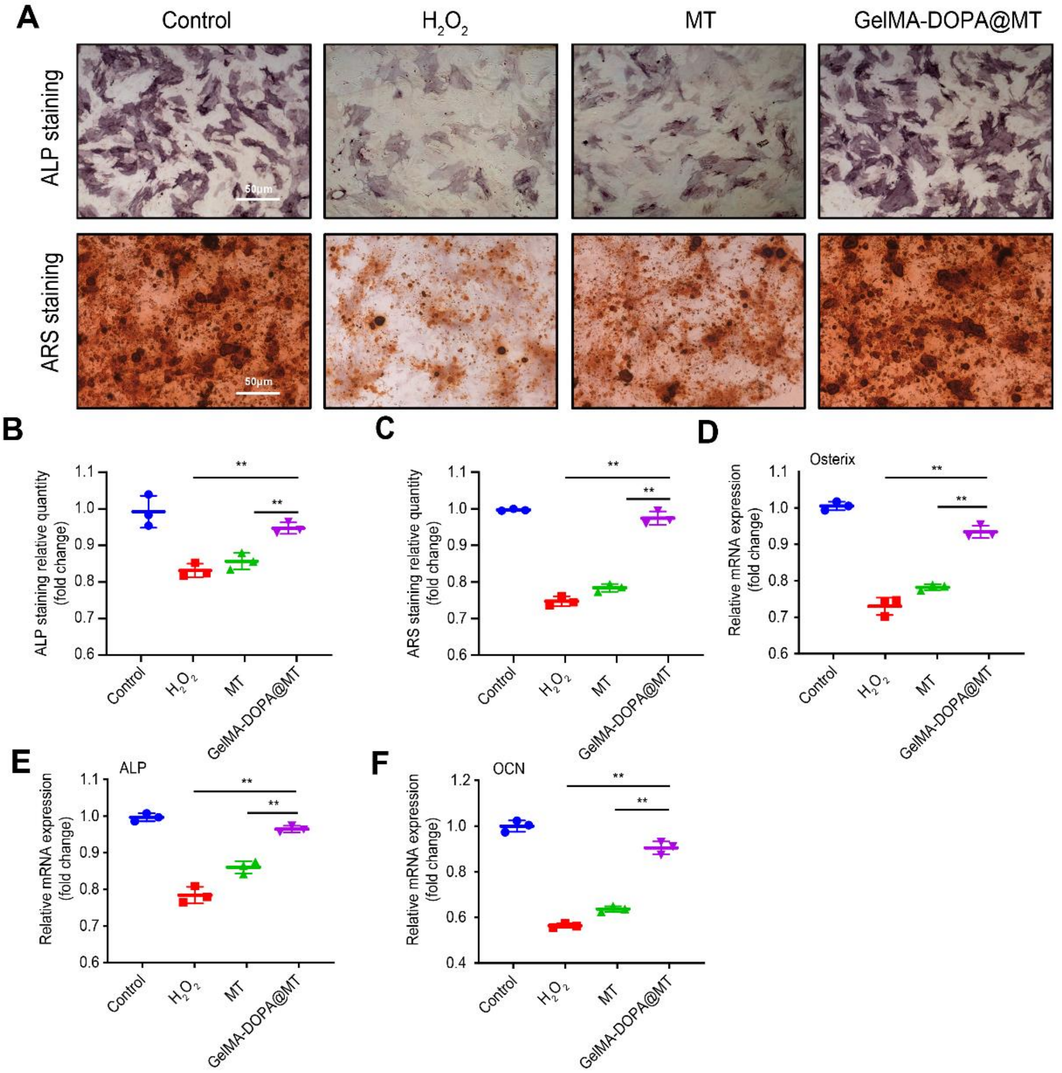

Figure S1. GelMA-DOPA@MT promotes the differentiation of osteoblasts in vitro（BMSC）. A) Representative images showing ARS and ALP staining, with red and black arrows indicating calcium nodules. Scale bar: 50 μm. B & C) Quantitative analysis of ARS and ALP staining. n = 3 per group, * $p < 0.05$, ** $p < 0.01$, vs. the GelMA-DOPA@MT group. D) qPCR analysis of the expression of the osteogenic genes Osterix, ALP and OCN in the experimental group, n = 3 per group, * $p < 0.05$, ** $p < 0.01$, vs. the GelMA-DOPA@MT group.

Well ID	Name	Well	450	Count	Mean	Std Dev	Well ID	Name	Well	450	Count	Mean	Std Dev	Well ID	Name	Well	450	Count	Mean	Std Dev
GelMA	1DAY	A1	0.321	5	0.3054	0.022832	GelMA	3DAY	C4	0.654	5	0.6624	0.029134	GelMA	7DAY	C4	2.52	5	2.6264	0.358914
		A2	0.289						C5	0.622						C5	2.887			
		A3	0.275						C6	0.698						C6	2.042			
		A4	0.312						C7	0.656						C7	2.873			
		A5	0.33						C8	0.682						C8	2.81			
GelMA-COOH		B1	0.319	5	0.3088	0.00795	GelMA-COOH		D4	0.671	5	0.66	0.01063	GelMA-COOH		D4	2.519	5	2.581	0.334452
		B2	0.299						D5	0.671						D5	2.909			
		B3	0.31						D6	0.656						D6	2.585			
		B4	0.313						D7	0.647						D7	2.833			
		B5	0.303						D8	0.655						D8	2.059			
GelMA-DOPA		C1	0.298	5	0.2998	0.01033	GelMA-DOPA		E4	0.621	5	0.6484	0.029712	GelMA-DOPA		E4	2.814	5	2.5938	0.19585
		C2	0.315						E5	0.688						E5	2.684			
		C3	0.299						E6	0.619						E6	2.387			
		C4	0.286						E7	0.667						E7	2.698			
		C5	0.301						E8	0.647						E8	2.386			
GelMA-DOPA@MT		D1	0.312	5	0.302	0.011225	GelMA-DOPA@MT		F4	0.659	5	0.657	0.017607	GelMA-DOPA@MT		F4	2.786	5	2.645	0.306976
		D2	0.302						F5	0.665						F5	2.108			
		D3	0.288						F6	0.681						F6	2.765			
		D4	0.294						F7	0.643						F7	2.873			
		D5	0.314						F8	0.637						F8	2.693			

Table S1. The quantitive results of CCK8 staining.
